# Limbal stem cells carried by a four-dimensional -printed chitosan-based scaffold for corneal epithelium injury in diabetic rabbits

**DOI:** 10.3389/fphys.2024.1285850

**Published:** 2024-06-03

**Authors:** Mengyuan Wang, Kaibin Liu, Xiaomin Wang, Zhen Shang, Yiming Liu, Nailong Pan, Xueqing Sun, Wenhua Xu

**Affiliations:** ^1^ Institute of Regenerative Medicine and Laboratory Technology Innovation, Qingdao University, Qingdao, China; ^2^ Department of Thoracic Surgery, Shanghai Jiao Tong University Affiliated Sixth People’s Hospital, Shanghai, China

**Keywords:** cornea, diabetes, stem cells, tissue-engineering scaffold, mesenchymal stem cells, regeneration

## Abstract

**Methods:** Herein, we obtained and characterized deltaN p63- and adenosine triphosphate-binding cassette subfamily G member 2-expressing limbal stem cells (LSCs). Chitosan and carboxymethyl chitosan (CTH) were cross-linked to be an in situ thermosensitive hydrogel (ACH), which was printed through four-dimensional (4D) printing to obtain a porous carrier with uniform pore diameter (4D-CTH). Rabbits were injected with alloxan to induce diabetes mellitus (DM). Following this, the LSC-carrying hydrogel was spread on the surface of the cornea of the diabetic rabbits to cure corneal epithelium injury.

**Results:** Compared with the control group (LSCs only), rapid wound healing was observed in rabbits treated with LSC-carrying 4D-CTH. Furthermore, the test group also showed better corneal nerve repair ability. The results indicated the potential of LSC-carrying 4D-CTH in curing corneal epithelium injury.

**Conclusion:** 4D-CTH holds potential as a useful tool for studying regenerative processes occurring during the treatment of various diabetic corneal epithelium pathologies with the use of stem cell-based technologies.

## 1 Introduction

Diabetes mellitus (DM) is a common and fatal metabolic disease, with currently approximately 451 million (Cho et al., 2018) cases and an increasing incidence rate over the years (Whiting et al., 2011). Approximately 46%–64% of patients with DM acquire diabetic keratopathy (Schultz et al., 1981). Diabetic keratopathy is the most frequent condition among human corneal diseases and has garnered worldwide clinical attention owing to its potential threat of serious corneal epithelial disturbances. DM can increase the susceptibility to spontaneous corneal traumas, such as epithelial erosions and ulcerations (Whiting et al., 2011; Wang et al., 2012) In patients with DM, any injury/trauma to the corneal epithelium takes longer to heal and epithelial lesions persist for a prolonged duration (Zagon et al., 2006). Patients with diabetic keratopathy are more prone to corneal surgery because of DM-associated complications, such as cataracts and glaucoma.

Stem cells are primarily responsible for the physiological and reparative regeneration of tissues (Yamanaka, 2020). Limbal stem cells (LSCs) are a kind of stem cells that originate from mesoderm and can be slowly renewed to repair cornea (Pellegrini et al., 1999). The limbus is a narrow area between an optically clear cornea and opaque sclera (Le et al., 2018), and LSCs present in the basal epithelial layer play an important role in the physiological and reparative regeneration of the corneal epithelium (Wilson et al., 2020).

Compared with three-dimensional (3D) printing, four-dimensional (4D) printing includes the fourth dimension of time; only stimulable smart materials can be used for 4D printing (Mitchell et al., 2018). 4D printing materials can produce different deformations after printing according to different external stimuli (such as light temperature, etc.) (Lu et al., 2021). Through 3D printing technology and memory materials, the shape and properties of 4D-printed materials can be changed over time via human intervention or spontaneous changes (Ionov, 2018). Owing to the properties of smart materials, 4D bioprinting may also provide a potential strategy to create scaffolds that can mimic the structural features of normal tissues (Li et al., 2021).

Chitosan, a natural polysaccharide, is extracted by N-deacetylation of chitin, which is the most abundant polymer on the earth after cellulose (Rinaudo, 2006). Moreover, chitosan is a Food and Drug Administration-approved polymer with high mucoadhesive properties that have been explored for biomedicines (Giri et al., 2012; Eivazzadeh-Keihan et al., 2021). Previously, we developed *in situ* hydrogels on the wound surface by the direct cross-linking of sodium alginate and CTH (Xu et al., 2019); however, the results of scanning electron microscopy showed that the prepared ACH surface had irregularities. The network structure with different pore sizes can directly reduce the loading efficiency of LSCs. Therefore, developing a carrier with uniform pore size and tunable shape to improve the transfer rate of cells and the degree of local adhesion of defects is crucial for improving tissue damage repair.

**FIGURE 1:**
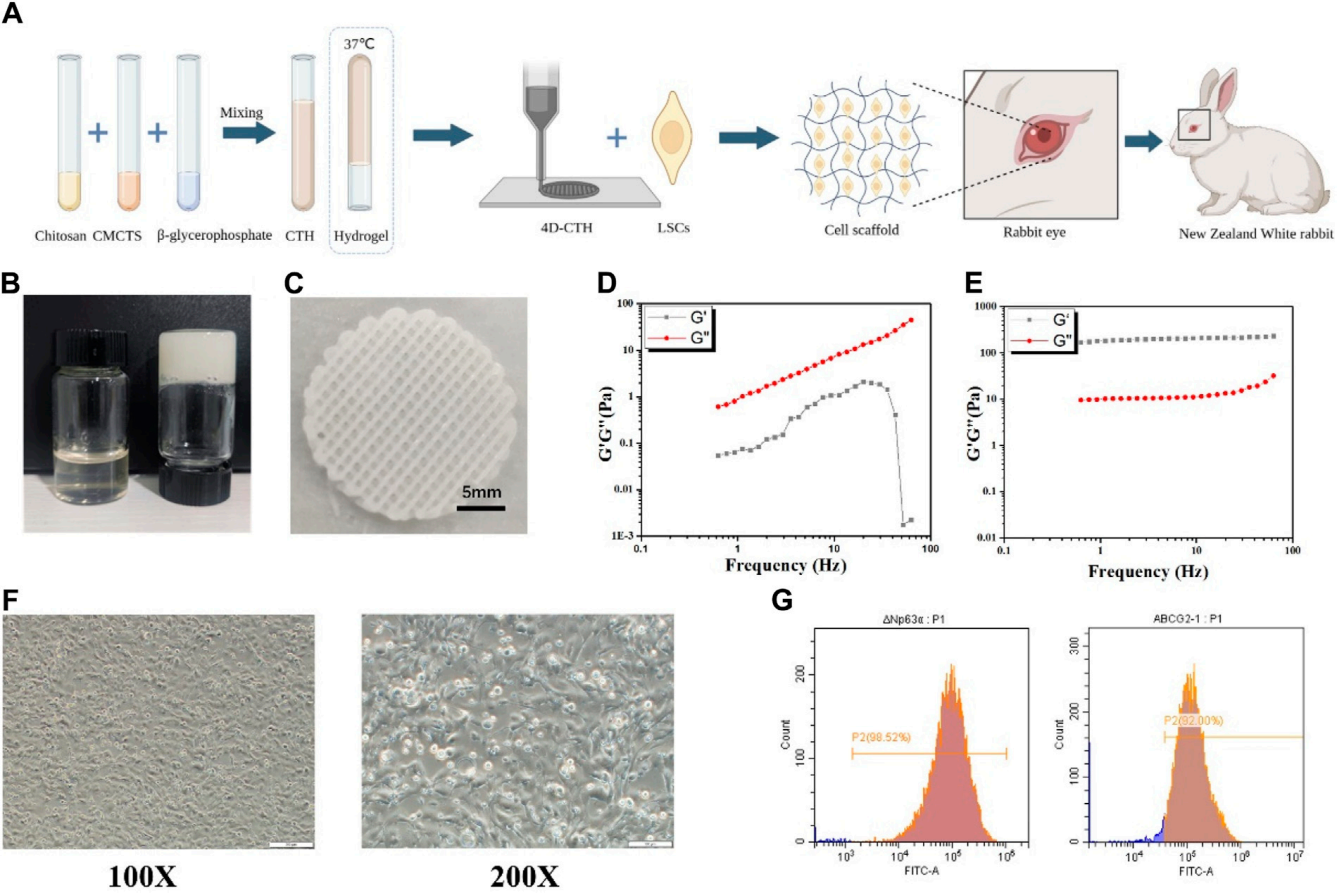
Four-dimensional (4D)- carboxymethyl chitosan (CTH) preparation and morphology of rabbit limbal stem cell (LSC) identification **(A)** Schematic diagram of the preparation process of CTH and the effect of loading LSCs in combination with 4D-CTH on corneal damage in diabetic rabbits **(B)** The thermoreversible sol-gel transition of the CTH between 4°C (left) and 37°C (right) **(C)** The structure of CTH prepared via 4D printing; **(D)** Storage modulus and loss modulus of CTH in 23°C measured in the frequency range of 0.1–100 Hz **(E)** Storage modulus and loss modulus of CTH in 37°C measured in the frequency range of 0.1–100 Hz; **(F)** Morphological observation of rabbit LSCs (P1); scale bar: 200 µm (left); scale bar: 100 µm (right) **(G)**) Flow cytometry detection of extracted LSC surface marker molecules; DeltaN p63 (left); adenosine triphosphate-binding cassette subfamily G member 2 (right).

Rabbits are the preferred animal model for ophthalmological research owing to their special advantage over other model animals because of their relatively larger eye size compared with those of rats or mice (Jiang et al., 2021; Khorolskaya et al., 2021; Zhong et al., 2021). Therefore, herein, we isolated rabbit LSCs, characterized them using multipotent stem cell-specific markers, and established an alloxan-induced diabetes model in New Zealand white rabbits. Additionally, we prepared a novel stem cell carrier with uniform pore size and adjustable shape, using two types of natural marine organism extracts and 4D bioprinting technology to improve the LSC loading rate and repair effect on corneal damage (Figure 1A).

## 2 Material and method

### 2.1 Prepare of 4D-CHT scaffold material

The scaffold was synthesized following the methodology of a previous study (Xu et al., 2021). The major steps involved in the synthesis process were as follows: chitosan (0.2 g) was dissolved in 4.5 mL of 0.1 mmol/L acetic acid solution under aseptic conditions and stirred for 10 h. Following this, the solution was mixed with CTH (0.2 g) and β-sodium glycerophosphate (0.8 g) dissolved in 4.5 and 1.0 mL of double distilled water, respectively, and the formed hydrogel was printed into a homogeneous carrier with a pore size of 200 µm using 4D printing technology at a low temperature (Bio-architect^®^ SR, Regenovo, China). The printed materials were freeze-dried in a vacuum and used as carrier scaffolds for cell transplantation.

#### 2.1.1 Rheological properties of 4D-CTH scaffold materials

The hydrogel was fabricated into 10-mm-diameter and 1-mm-thick slices, which were then placed on the support plate of the rheometer (Anton Paar). The storage modulus and loss modulus of the gel slices were measured at a shear frequency of 0.1–100 Hz and strain of 1%. Data analysis and drawings were carried out using OriginPro 9.1.

### 2.2 Cell culture

#### 2.2.1 Primary culturing of the LSCs

The rabbits were sacrificed by cervical dislocation, and the whole eyeballs were removed, washed several times with phosphate-buffered saline (PBS) (Dalian Meilun Biotechnology Co., Ltd., China), and treated with 400 U/mL penicillin or streptomycin for 30 min. Under a stereomicroscope, a 2-mm circular tissue was excised from the gray junction between the conjunctiva. The obtained corneal rings were digested overnight with 1.2 u/mL dispase II (Beijing Solaibao Technology Co., LTD., China). Under a stereomicroscope, the corneal epithelium was removed using ophthalmic tweezers and digested with pancreatic enzymes for 15 min. After digestion, the tissues were placed in the culture flasks in a 37°C CO_2_ incubator. Cell migration was observed daily using an inverted microscope. The obtained LSCs were incubated on culture plates for 3 days. LSCs were cultured with mesenchymal stem cell culture medium (Shenzhen Dakowei Biotechnology Co., LTD., China) containing 5% serum substitute (EliteCell Inc., United States) and 1% penicillin-streptomycin solution (Dalian Meilun Biotechnology Co., Ltd., China).

#### 2.2.2 Identifying LSC

The second-generation LSCs were identified using the following two methods: cell morphology observation and flow cytometry. When the cell density reached 60%–80%, the cells were treated with trypsin (Dalian Meilun Biotechnology Co., Ltd., China) for 1.5 min. Following this, the culture was centrifuged at 1,000 rpm for 5 min, and the pellet was washed with PBS (Dalian Meilun Biotechnology Co., Ltd., China). The pellet was blocked with bovine serum albumin (BSA) cell-staining buffer (Dalian Meilun Biotechnology Co., Ltd., China) for 20 min, and then washed twice with the cell-staining buffer. The cells were incubated with anti-delta nP63 (Santa Cruz Biotechnology, Inc., United States, 1:250) and anti-adenosine triphosphate-binding cassette subfamily G member 2 (ABCG2) antibodies (Santa Cruz Biotechnology, Inc., United States, 1:200) at 4°C for 30 min, and then were washed twice with cell-staining buffer. Following this, the cells were incubated for 30 min with Dylight 488-conjugated AffiniPure goat antimouse immunoglobulin G (IgG, H + L) (Boster Biological Technology, China, 1:500) without light exposure. Thereafter, the cells were resuspended in 200 μL cell-staining buffer, and the sample was subjected to flow cytometry (Beckman Coulter, Inc., United States) for detecting LSCs.

### 2.3 Evaluation of cytocompatibility of 4D-CTH

#### 2.3.1 Fluorescent scratch test

LSCs were inoculated in six-well plates with sterile 4D-CTH in the experimental group and no treatment in the control group, and incubated overnight at 37°C in a cell culture incubator. After the cells were overgrown, they were scratched with the tip of pipette, and the detached cells were removed by washing with serum-free medium. Next, 500 µL of 2 µM calcein-acetoxymethyl (AM) (Dalian Meilun Biotechnology Co., Ltd., China) was added to each well at 0, 12, and 24 h, and the plate was incubated at room temperature for 15 min. The staining solution was discarded, and PBS was added to the cells. The image of scratch healing was taken using an inverted fluorescence microscope. The ImageJ software was used to quantify the wound width and calculate the wound healing rate.

#### 2.3.2 Staining of AM/PI

LSCs were inoculated in twelve-well plates with sterile 4D-CTH in the experimental group and no treatment in the control group, and incubated overnight at 37°C in a cell culture incubator. Preparation of Calcein-AM (AM) and propidium iodide (PI) staining working solution: take out the stock solution of AM and PI in advance, put it at room temperature to equilibrate for 30 min, add 5 μL of AM and 5 μL of PI stock solution in 10 mLPBS, and get the concentration of 2 μM of AM and 8 μM of PI. AM/PI staining working solution. After the LSCs and 4D-CTH were co-incubated for 24, 48 and 72 h, the cells were gently washed with PBS for 2–3 times to ensure the removal of active esterases present in the medium, and 250 μL of the pre-configured AM/PI staining working solution was added to each well, and the cells were incubated for 15 min at room temperature and protected from light. Discard the AM/PI staining working solution, and then take pictures of cell staining with an inverted fluorescence microscope. The fluorescence intensity of living and dead cells was calculated by ImageJ software.

### 2.4 Corneal epithelial wound healing

#### 2.4.1 Induction and management of diabetes mellitus

New Zealand white rabbits were purchased from Qingdao Kangda Aibo Biotechnology Co., Ltd. (SCXK 2021 0003). Alloxan monohydrate (Sigma Aldrich Chemical, Saint Louis, MO, United States) was dissolved in sterile normal saline to achieve a concentration of 5% (W/V), and 100 mg/kg alloxan was immediately administered intravenously via the marginal ear vein for 2 min, and the rabbits were allowed to recover from anesthesia. To avoid mortalities during the hypoglycemic phase, alloxan was administrated to nonfasted animals, and food and water were provided to animals immediately after injection. At 4, 8, and 12 h after alloxan injection, if hypoglycemia (<70 mg/dL blood sugar level) occurred, 10 mL of glucose (5% W/V) (Sigma Aldrich Chemical, Saint Louis, MO, United States) was applied under the skin, and 20% glucose water was provided for 1–2 days to prevent blood sugar shock. One week after the first alloxan injection, rabbits with a blood glucose level of <300 mg/dL received the second dose of acid (100 mg/kg) intravenously to maintain the blood glucose levels at >300 mg/dL. A glucose meter (Lifespan, Inc., Milpitas, CA) was used to measure the blood sugar levels daily.

#### 2.4.2 Construction of corneal mechanical injury model

The New Zealand large white rabbits were fixed with a frame and anesthetized by injecting 1%/kg pentobarbital sodium solution. The eyelids and their surroundings were disinfected with a cotton swab dipped in 75% alcohol, and then the ocular surface of the rabbit was anesthetized topically with 0.5% lidocaine hydrochloride. An 8-mm-diameter corneal ring drill was used to determine the extent of epithelial scraping. The scraping area was gently wiped with a cotton swab dipped in 75% alcohol, and the epithelium was scraped using an Algerbrush II (Alger Inc. United States), and an iris spatula was used to smoothen the scraped area.

#### 2.4.3 Cell transplantation and *in vivo* examinations

The rabbits were randomly divided into the following three groups, with three rabbits in each group: the control group, epithelium-scraped ocular surface rinsed with PBS only; the cell group, LSCs added dropwise to the damaged ocular surface; and the 4D-CTH treatment group, a 4D-CTH carrier was used to transfer LSCs to the damaged ocular surface. During treatment, the upper and lower eyelids of the rabbits were sutured and 0.3% ofloxacin eye drops were routinely administered daily to prevent infections. The sutures were removed from the eyes at postoperative 1 day, and the healing of the ocular surface and trauma in each group was observed and photographed daily. Simultaneously, the eyes of the rabbits from each experimental group were stained with 0.2 µL of sodium fluorescein, and photos were captured under the blue light. The corneal thickness was measured using a handheld corneal thickness meter (PachPen, Accutome, Inc., United States). The intraocular pressure was monitored using a tonometer (Accupen, Accutome, Inc., United States).

#### 2.4.4 Histological observations microscope imaging

On day 10 after the surgery, the rabbits were euthanized, their eyeballs were removed, and the corneas were stripped. The corneas were divided into two parts along the midline. One part was fixed with 10% formalin (Jinqiao Co., Ltd., Beijing, China), embedded in paraffin. After the paraffin wax is cut into thin slices, it is soaked in xylene (Dalian Meilun Biotechnology Co., Ltd., China) three times, each time for 10 min. Put in anhydrous ethanol, 95% ethanol and 75% ethanol respectively, and soak each concentration twice for 3 min each time. The slices were rinsed in distilled water for 1 min and then soaked in PBS (Dalian Meilun Biotechnology Co., Ltd., China) buffer for 3 min. Dye with hematoxylin solution (Jinqiao Co., Ltd., Beijing, China) for 5 min, then rinse with distilled water to remove surface float. Soak in the differentiation solution for 10–30 s and examine the degree of differentiation under a microscope. Dye with eosin solution (Jinqiao Co., Ltd., Beijing, China) for 30 s to 2 min, then quickly remove the liquid from the surface. The slices were dehydrated in a solution containing 95% ethanol for 2–3 s, then dehydrated with anhydrous ethanol three times for 5–10 s each time. Put the slices into xylene and transparent for 3 times, 2 min each time, and seal the slices with neutral gum; After the slices were dried, they were photographed using an inverted microscope.

#### 2.4.5 Immunofluorescence staining

The stripped corneas were embedded in the Tissue-Tek OCT complex (Jinqiao Co., Ltd., Beijing, China). Further, these frozen corneas were cut using a frozen sectioning machine, fixed in 4% paraformaldehyde (Dalian Meilun Biotechnology Co., Ltd., China) for 15 min, and washed thrice with PBS for 5 min each time. The cells were then blocked with a blocking buffer (5% BSA +0.3% Triton X-100) (Dalian Meilun Biotechnology Co., Ltd., China) for 1 h at room temperature. Next, the blocking buffer was discarded, and the tissues were incubated with antibodies against cytokeratin (CK) 3 (Immuquest, United Kingdom, 1:200), α-smooth muscle actin (Sigma-Aldrich, MO, United States, 1:400), tumor necrosis factor (TNF)-α (Novus Biologicals, CO, United States, 1:100), and Ki-67 (Novus Biologicals, CO, United States, 1:500) overnight at 4°C, followed by incubation in the night with the F-labeled goat anti-rabbit IgG secondary antibody (Cell Signaling Technology, Danvers, MA, United States, 1:1,000) for 2 h at room temperature. Finally, the tissues were sealed with DAPI-containing antifluorescent attenuation sheets (Dalian Meilun Biotechnology Co., Ltd., China). Images were captured using an inverted fluorescence microscope.

#### 2.4.6 *In vivo* organ toxicity experiment

After the execution of the New Zealand Large White rabbits, they were divided into PBS and 4D-CTH + LSCs groups. Further, their livers, kidneys, and spleens were removed, weighed, fixed, and HE stained to observe any pathological changes in the morphology of the organs.

### 2.5 Statistical analysis

Experimental data were statistically analyzed using the SPSS v.19.0 software, and quantitative data were expressed as the mean ± standard deviation (X ±SD). After confirming the normal distribution of the data, the mean difference was evaluated with a single-factor analysis of variance or *t*-test. Results were considered statistically significant at *p* < 0.05.

## 3 Result

### 3.1 Physical properties of 4D-CTH gels

The prepared CTH exhibited a flowing sol state at 4°C and solidified gel state when the temperature increased to approximately 37°C (Figure 1B). A self-modified biological printer was used to prepare 4D-CTH that could be deformed in response to temperature changes. According to the measured rabbit cornea data, a 3D model of 4D-CTH was constructed by matching software, and the temperature-sensitive CTH was injected into the biological printer to print the 4D-CTH, which could become a gel at 37°C under the control of low temperature and specific parameters. The 4D-CTH appeared white with a porous structure and uniform thickness (Figure 1C). The viscoelastic behavior of the chitosan-based thermosensitive gel was measured by dynamic oscillation rheology, and the function diagrams of the storage modulus (G′) and loss modulus (G″) as a function of frequency were drawn. The results show that in the frequency range of 0.1–100 Hz and at 23°C, the storage modulus and loss modulus of CTH increase with the increase of frequency, and the storage modulus is always smaller than the loss modulus, and it is in the liquid state (Figure 1D). However, at 37°C, the storage modulus of CTH was always larger than the loss modulus and remained in the solid state (Figure 1E). As discussed in the viscoelastic behavior measurement, the results of mechanical properties indicated that the prepared carrier had excellent thermo-sensitive hydrogel properties.

### 3.2 Identification of LSCs and cytocompatibility assays of 4D-CTH

A recent study has shown that stem cells can regulate the microenvironment of chronic wounds by secreting anti-inflammatory and growth factors, ultimately promoting wound healin (Li et al., 2022). Thus, LSCs were initially extracted in the present study. The first-generation LSCs were observed under an inverted microscope, which showed that the cells adhered to the wall in a typical oblong shape (Figure 1F). Cell surface markers were identified by subjecting the second-generation cells to flow cytometry. DeltaN p63 and adenosine triphosphate-binding cassette subfamily G member 2 were detected by flow cytometry, and the isolated cells matched the immunophenotype of LSCs (Figure 1G).

**FIGURE 2:**
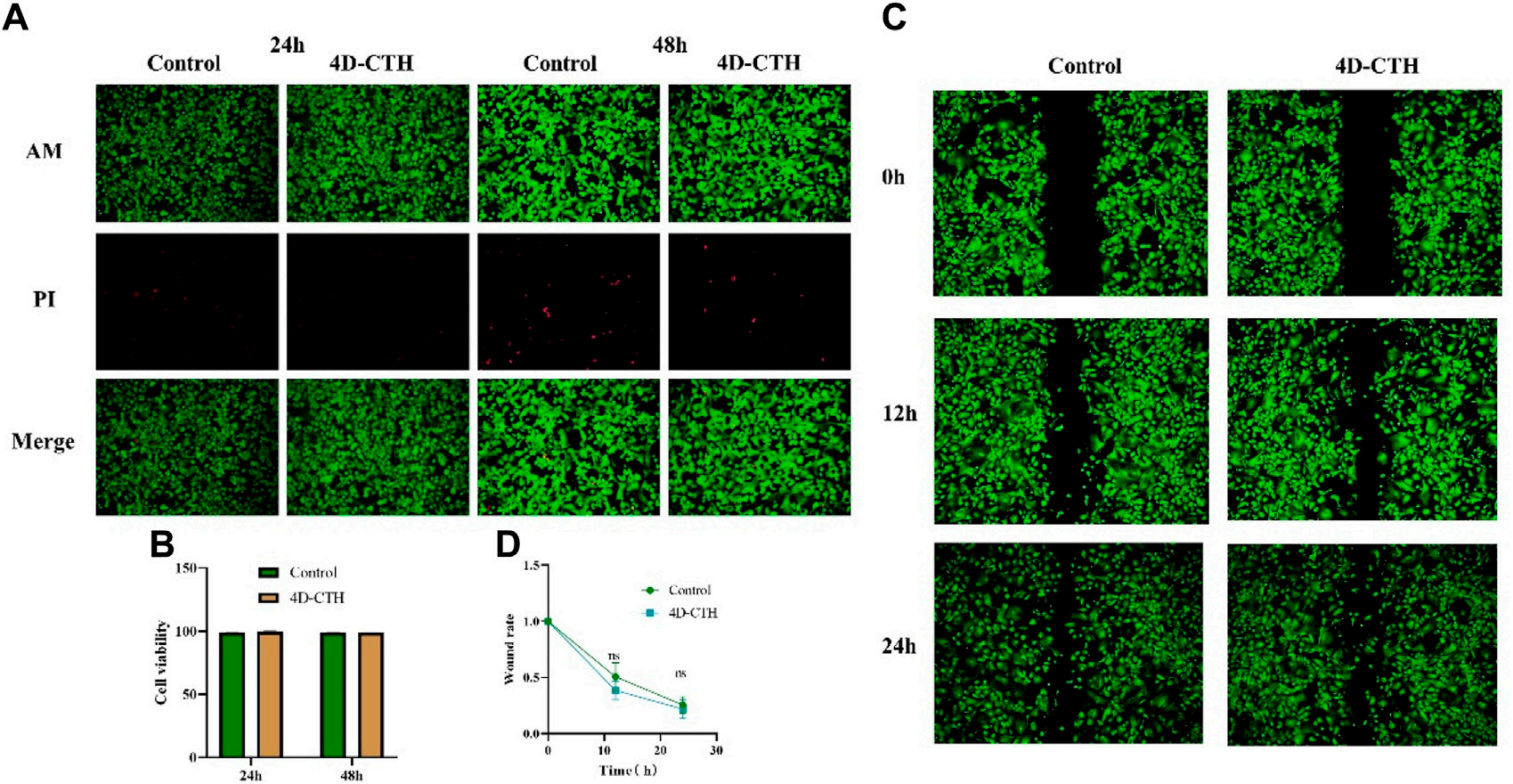
Biocompatibility of four-dimensional (4D)- carboxymethyl chitosan (CTH). **(A)** Images of live/dead cell staining (acetoxymethyl [AM]/propidium iodide [PI]) under the inverted microscope after 24 and 48 of co-incubation with 4D-CTH; scale bar: 200 μm **(B)** Statistical analysis of cell viability; **(C)** Images of wound healing experiments at 12 and 24 h after calcein (AM) staining of the control and 4D-CTH groups; scale bar:500 μm; **(D)** Statistical analysis of scratch healing rate.

However, the direct injection of LSCs into wounds reduces the bioactivity of these cells. A unique 3D porous structure of a hydrogel can continuously release bioactive substances embedded in the hydrogel to maximize the retention of the bioactivity of mesenchymal stem cells (MSCs) (Hilal et al., 2023). Therefore, the 4D-CTH effect on LSC activity was evaluated. Calcein AM/PI staining was performed to assess the 4D-CTH effect on cell viability, which showed that after co-culturing LSCs with 4D-CTH for 24 and 48 h the cells in the 4D-CTH and control groups were well adhered to the wall, and the typical elongated round cell morphology of LSCs was maintained (Figure 2A). Cell viability was greater than 90% for both groups, and the difference between the two groups was not statistically significant (*p* > 0.05) (Figure 2B), suggesting that the unique porous structure of 4D-CTH provided a favorable environment for cell survival and facilitated cell growth. Similarly, because cell migration is crucial for wound healing, we examined the 4D-CTH effect on LSC migration ability. The results showed that 47.53% and 29.79% of the area was damaged at 12 h and 24 h, respectively, in the 4D-CTH group compared with 50.71%% and 25.53% of the area damaged in the control group (Figure 2C). Statistical analysis showed that the difference between the two groups was not statistically significant (*p* > 0.05) (Figure 2D), indicating that 4D-CTH did not affect cell migration.

### 3.3 Construction of a rabbit model of DM

**FIGURE 3:**
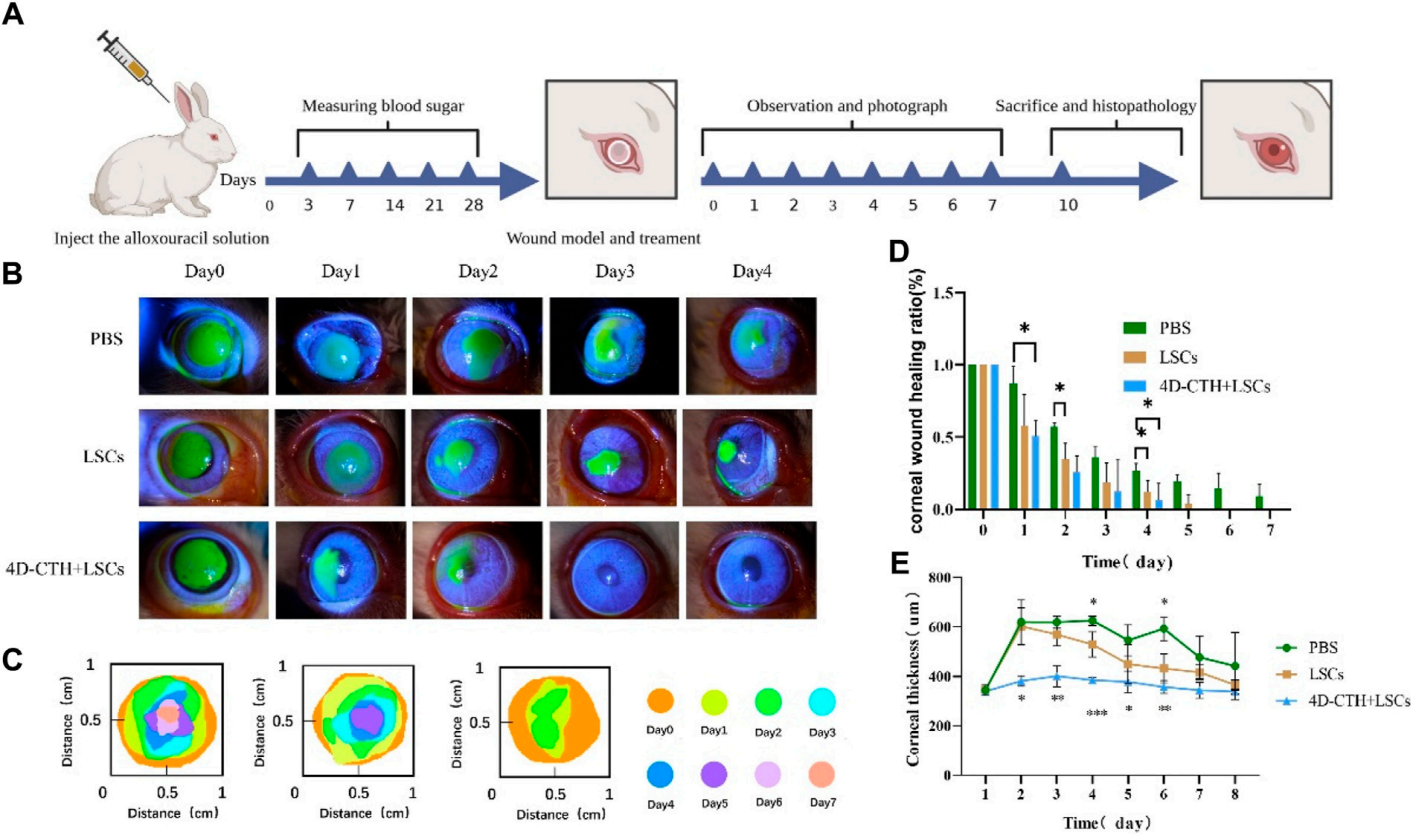
Effect of different treatments on corneal wound healing *in vivo*. **(A)** Graph of experimental progress of the Great White rabbit; **(B)** Cobalt blue light images of wound healing under different treatment conditions at 0, 1, 2, 3, and 4 days of wound healing **(C)** Schematic diagram of corneal wound area under different treatment conditions; **(D)** Results of the statistical analysis of the wound area at 0–7 days of wound healing under different treatment conditions; **(E)** Corneal thickness under different treatment conditions; *p < 0.05, **p < 0.01, **p < 0.01. ***p < 0.001.

The blood glucose levels and body masses of diabetic rabbits were measured 4 weeks after tetracosactide injection to establish a rabbit model of DM (Table 1). Rabbits with increased blood glucose levels (blood glucose level >13.9 mmol/L) were selected for diabetes modeling, which showed typical diabetic symptoms, including polyphagia and polyuria, and successfully modeled rabbits were used for the subsequent experiments (Figure 3A). Diabetic rabbit corneal epithelial injury models were established and divided into PBS, 4D-CTH, and LSC groups to observe under cobalt blue light after sodium fluorescein staining in order to evaluate the effect of each treatment on the repair of diabetic rabbit corneal wounds (Figure 3B). The quantitative analysis of the wound area showed that on the first postoperative day, the remaining wound areas observed in the PBS, LSC, and 4D-CTH + LSC treatment groups were 87.17%, 57.76%, and 50.86%, respectively, which indicated a significant healing effect of 4D-CTH + LSCs compared with that of PBS (*p* < 0.05). The wound areas observed on day 4 post-surgery in the PBS, LSC, and 4D-CTH + LSC treatment groups were 26.83%, 12.19%, and 6.61%, respectively, suggesting that the combination of 4D-CTH and LSCs promoted the healing of diabetic wounds to a certain extent, and the combination of the two had the same effect (*p* < 0.05) (Figures 3C, D). This combination provided a favorable microenvironment for wound healing. Finally, on day 7, the wound treated with the combination was completely healed (Supplementary Figure S1).

**TABLE 1:** Blood glucose in New Zealand white rabbits. (Mmol/L).

Time Number	Day3	Day7	Day14	Day21	Day28
1	26.1	22.9	22.4	24.3	27.9
2	21.8	22.2	21.2	20.8	17.2
3	16.4	18	23.9	21.9	24.2
4	20.4	19.8	17.1	23.9	25.4
5	32.8	28.8	31.7	29.6	29.8
6	21.9	22.9	22.4	19.3	20.4
7	24.9	23.2	28.6	24.4	24.3
8	25	24.9	28.5	32.5	30.4
9	24.4	23.5	27.2	25.8	29.6

The examination of the postoperative rabbits using a corneal thickness meter and tonometer showed that the treatment with 4D-CTH-encapsulated LSCs rapidly eliminated the corneal edema after injury and restored the normal corneal thickness (Figure 3E). Additionally, the postoperative intraocular pressure of the white rabbits in each group increased slightly; however, no significant difference was observed between the groups (Supplementary Figure S2).

### 3.4 HE staining

**FIGURE 4:**
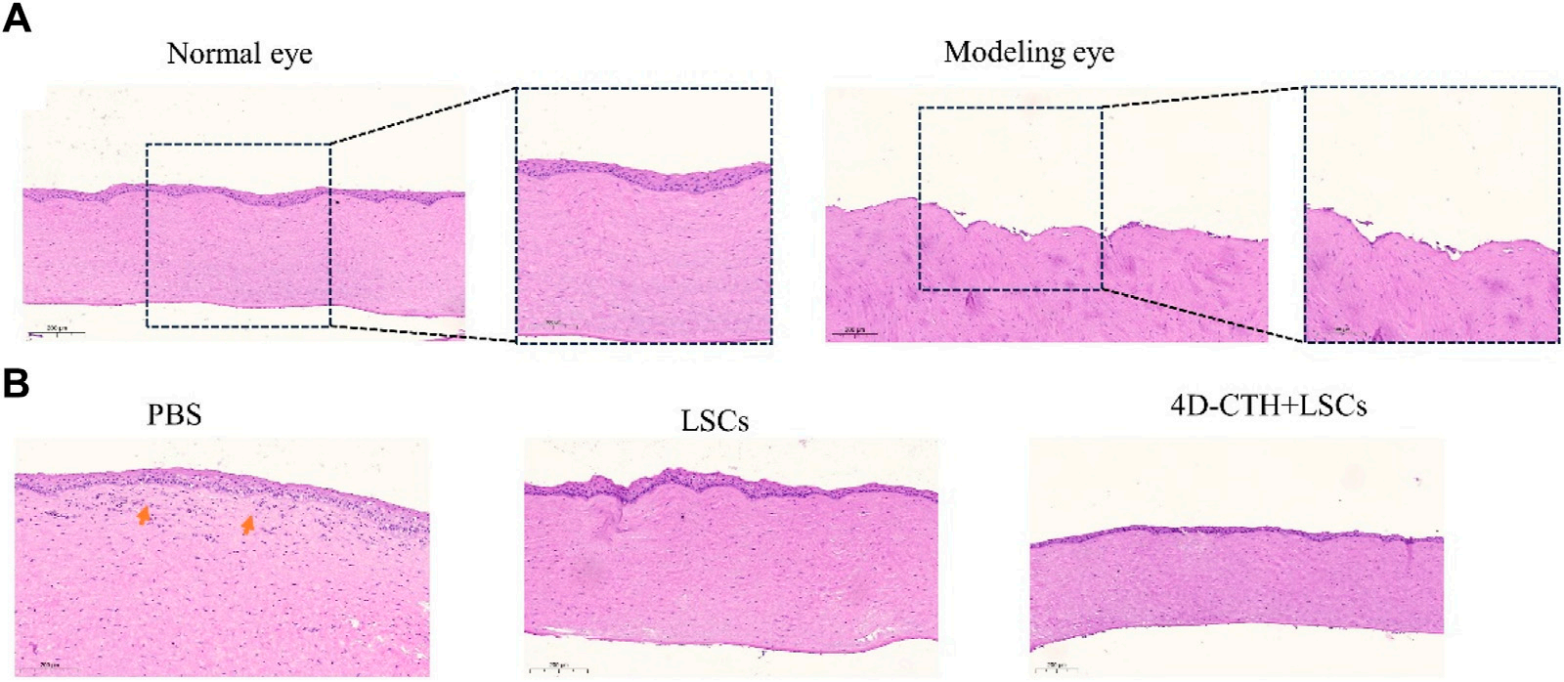
Assessment of corneal epithelial damage modeling and the degree of traumatic epithelialization. **(A)** Normal rabbit cornea and rabbit cornea after performing corneal epithelial scraping; scale bar: 200 μm (left); scale bar: 100 μm (right); **(B)** Representative HE-stained images of different treatment groups on the 10th day of wound healing; scale bar: 200 μm.

To verify whether the modeling of corneal epithelial scraping was successful, we performed the HE staining of the normal rabbit corneas and corneas after epithelial scraping. The results showed that the modeled corneal epithelium was completely scraped without any damage to the corneal stroma (Figure 4A).

HE staining performed to observe wound healing showed that on day 7 of wound healing, epithelialization was almost complete, and the thickness was closer to that of the normal cornea in the 4D-CTH + LSC group than in the PBS group, and fewer inflammatory cells (as indicated by the red arrows) were found in the stroma (Figure 4B). Therefore, we hypothesized that 4D-CTH and LSCs could synergistically promote epithelial regeneration and inhibit inflammation.

### 3.5 Tissue immunofluorescence staining analysis

**FIGURE 5:**
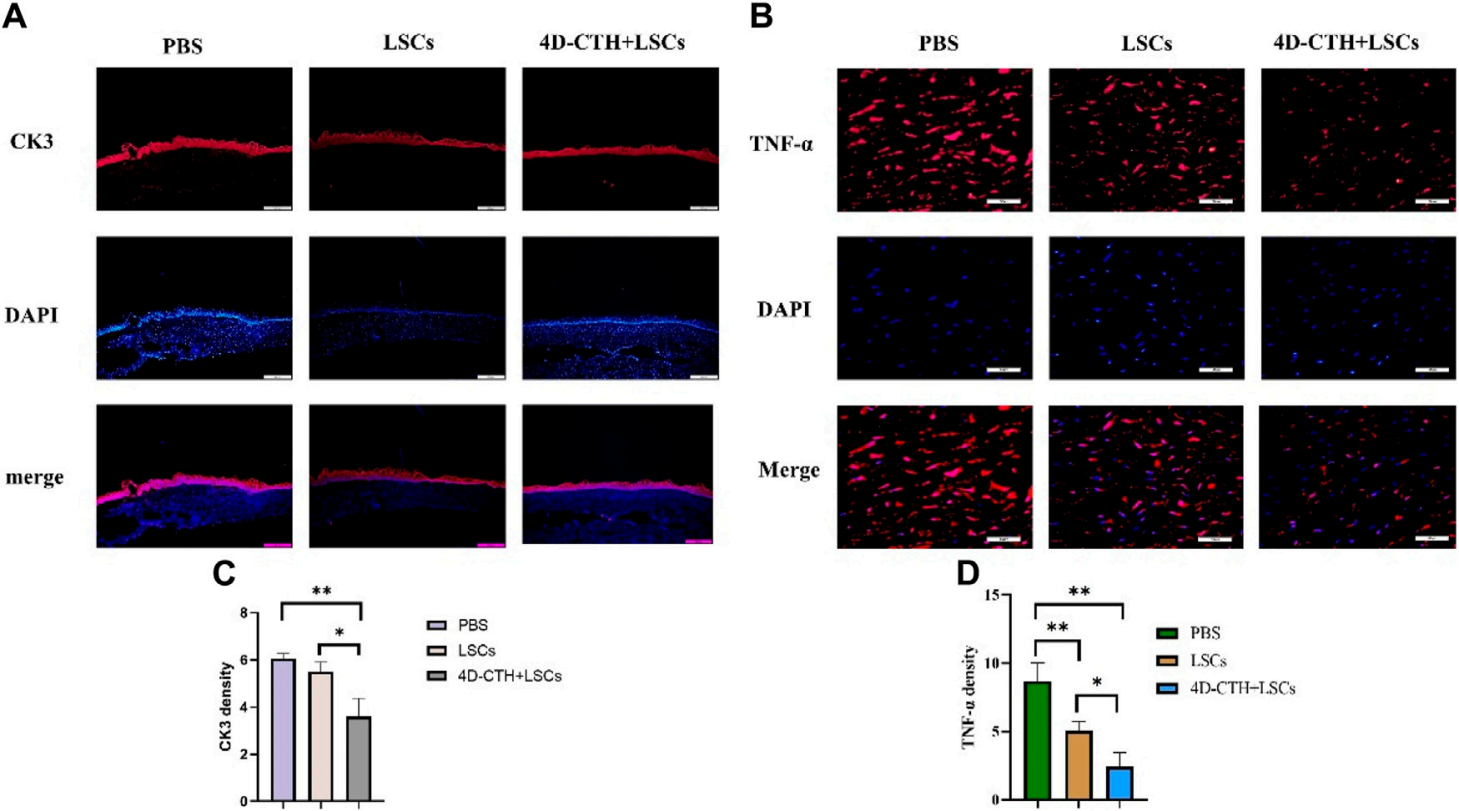
CK3 and TNF-α immunofluorescence staining to evaluate the effects of different treatments on wound epithelialization and inflammation occurrence. **(A)** At day 10, PBS, LSCs and 4D-CTH + LSCs groups were subjected to CK3 (red) immunofluorescence staining and DAPI (blue, nuclear stain) detection. Scale bar:500 μm **(B)** Immunofluorescence staining with TNF-α (red) and DAPI (blue, nuclear staining solution) was detected in PBS, LSCs, and 4D-CTH + LSCs groups on day 10, scale bar:100 μm **(C)** Quantitative analysis of the TNF-α fluorescence intensity on day 10 of corneal wound healing; **(D)** Quantitative analysis of the TNF- α fluorescence intensity quantitative analysis; *p < 0.05, * *p < 0.01.

To further determine the potential mechanism by which the combination of 4D-CTH and LSCs promoted wound healing, we examined the levels of various functional proteins during corneal wound healing. The rabbit corneal tissues were collected on day 10 after corneal injury repair and subjected to frozen sectioning and immunofluorescence staining, and CK3 was used as a marker of the corneal epithelial layer to detect the degree of healing of the corneal epithelium (Figure 5A). The results showed that the corneal epithelial cells in the 4D-CTH + cell group were tightly packed, whereas those in the control and LSC groups were sparse and loosely connected (*p* < 0.01) (Figure 5C). We hypothesized that the difference in the degree of epithelial fragmentation between the groups might be because the 4D-CTH scaffold provided a favorable environment for the cells to survive, allowing the newly generated corneal epithelial cells to be tightly connected and functionally more capable of protecting the internal tissues.

Because corneal thickness varied among the groups in the animal test and more inflammatory cells were observed in the PBS group in HE staining, we performed corneal immunofluorescence staining using the inflammatory marker TNF-α (Figure 5B). The results showed that TNF-α levels decreased significantly (*p* < 0.01) in the LSC and 4D-CTH + LSC groups than in the PBS group (Figure 5D). This indicated that 4D-CTH reduced the inflammatory response at the wound site by means of LSCs, thereby reducing corneal edema, maintaining normal corneal thickness, and thus protecting vision.

**FIGURE 6:**
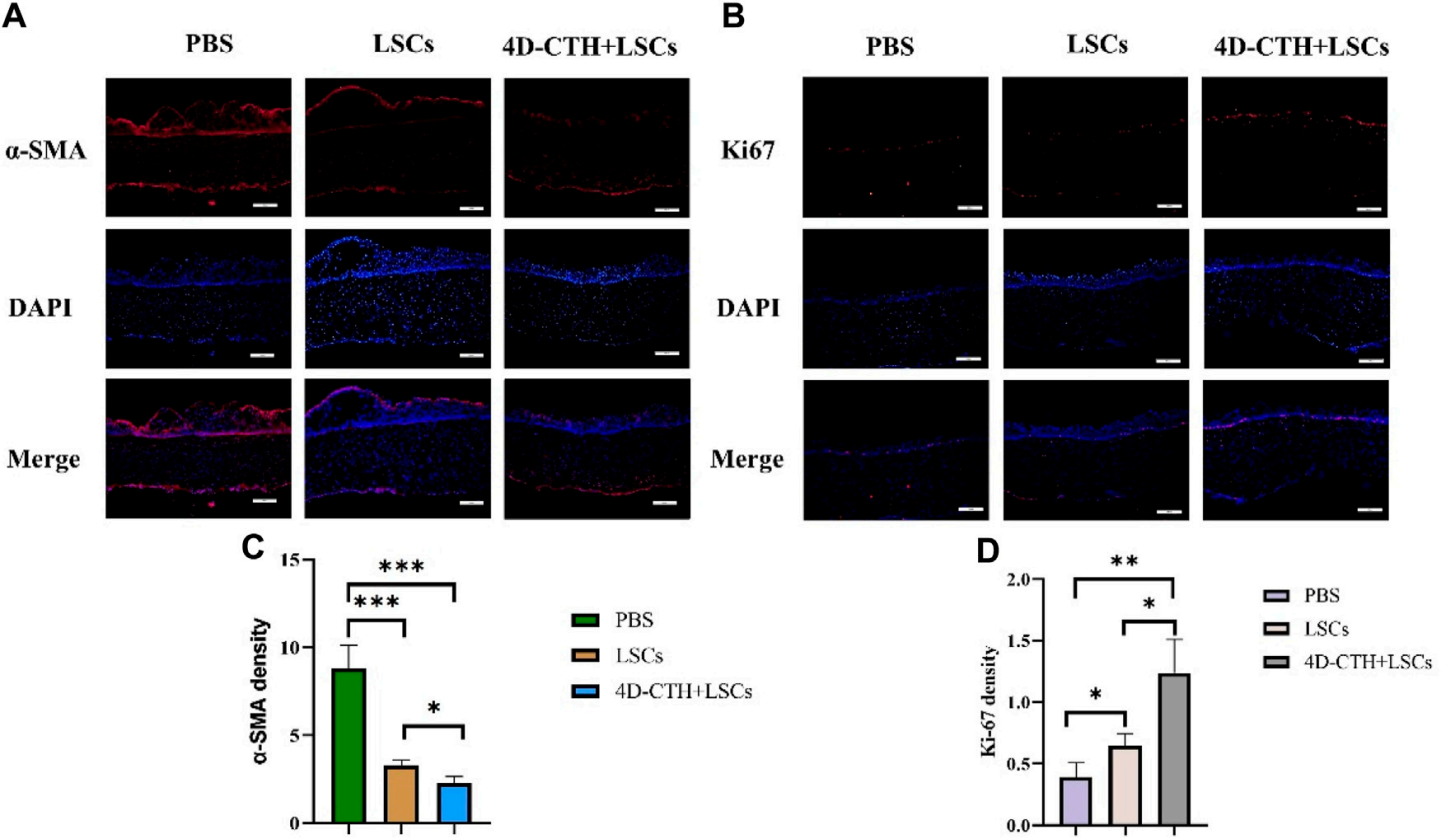
α-smooth muscle actin (SMA) and Ki67 immunofluorescence staining evaluated the effects of different treatments on scar formation and proliferation. **(A)** On day 10, α-SMA (red) immunofluorescence staining and DAPI (blue, nuclear staining solution) were performed in the PBS, LSC, and 4D- CTH + LSC groups; scale: 200 μm; **(B)** on day 10, PBS, LSC, and 4D-CTH + LSC groups were detected by Ki67 (red) and DAPI (blue, nuclear staining solution) immunofluorescence staining; scale of 200 μm; **(C)** quantitative analysis of α-SMA fluorescence intensity on day 10 of corneal wound healing; **(D)** quantitative analysis of Ki67 fluorescence intensity on day 10 of corneal wound healing; *p < 0.05, * *p < 0.01, * * *p < 0.001.

Wound contraction and scar formation are usually associated with cell migration. α-SMA can reflect cell migration to a certain extent, thus reflecting the state of scar formation after corneal injury. α-SMA levels decreased significantly in the LSC and 4D-CTH + LSC groups than in the PBS group (*p* < 0.001) (Figures 6A, C). The result indicated that the LSC and 4D-CTH + LSC groups promoted cell migration to a certain extent, which in turn led to irregular cell arrangement, poor light transmittance, and ultimately scar formation.

The immunofluorescence results showed that the levels of Ki67, a marker of cell proliferation, increased in the LSC and 4D-CTH + LSC groups than in the PBS group (*p* < 0.05 and *p* < 0.01, respectively) (Figures 6B, D). This suggested that the 4D-CTH and LSC combination could more effectively promote cell proliferation and accelerate wound healing.

**FIGURE 7:**
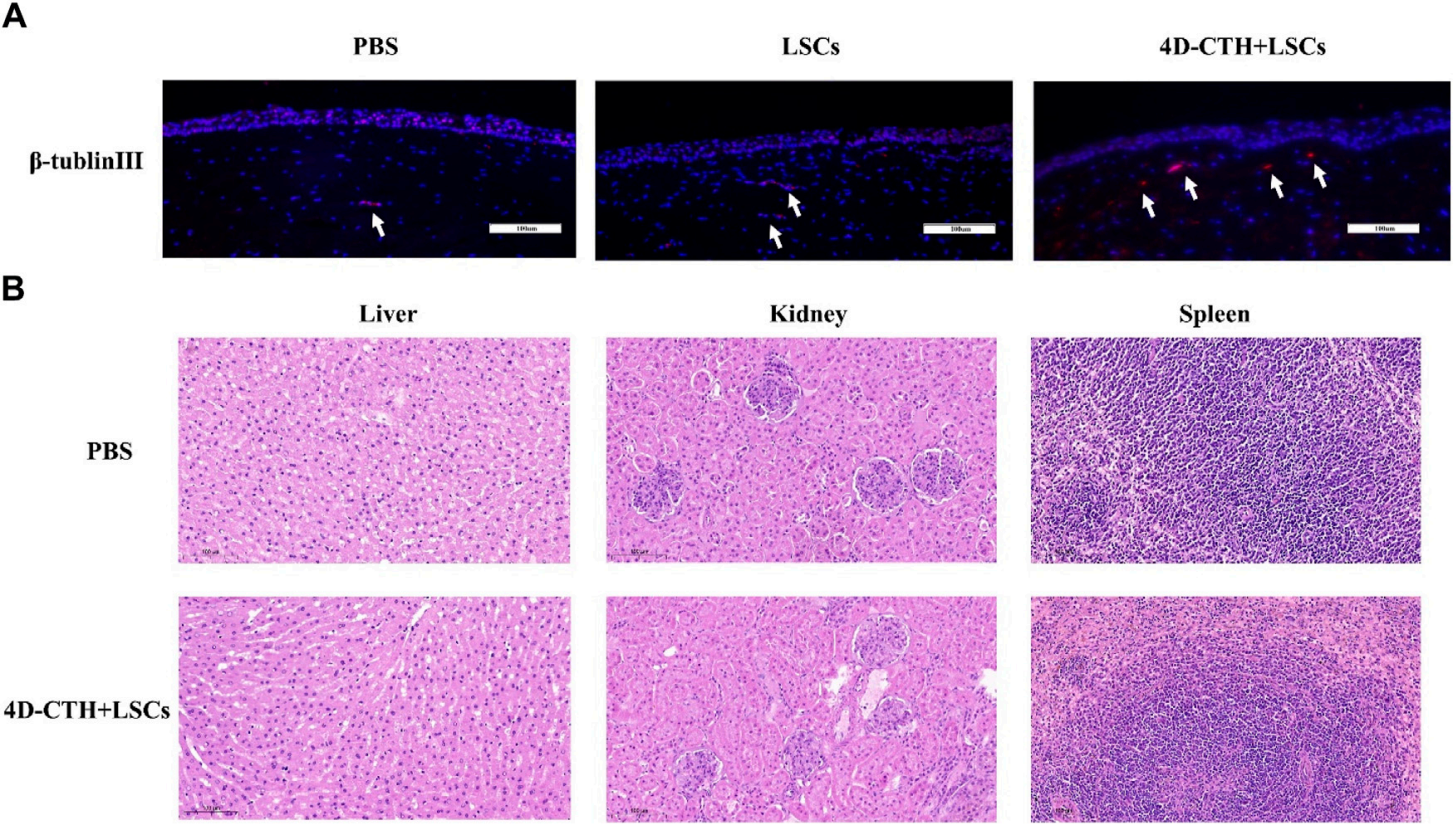
β-tublinⅢ immunofluorescence staining evaluated the effects of different treatments on nerve repair and organ toxicity analysis. **(A)** on day 10, PBS, LSC, and 4D-CTH + LSC groups were detected by β-tublinⅢ (red) and DAPI (blue, nuclear staining solution) immunofluorescence staining; scale of 100 µm; **(B)** Hematoxylin–eosin staining analysis of the liver, kidney, and spleen. Scale = 100 µm.

The high sugar environment associated with diabetes can damage peripheral nerves and disrupt the nutritional interaction between nerves and cells, thus delaying wound healing (Yu et al., 2022). In order to investigate the effect of LSCs on corneal nerve repair, we performed immunofluorescence staining experiments using the neural cell marker β-tubulin Ⅲ. The results of fluorescence staining of corneal tissue showed that the 4D-CTH + LSCs group showed a denser nerve distribution (Figure 7A). We speculate that the reason for this promotion of nerve repair may be due to the overall low inflammatory response and high cell proliferation in the injured area. This provides a suitable microenvironment for nerve cell growth, which further promotes nerve repair.

### 3.6 Organotoxicity

The liver, kidney, and spleen tissues of the control and 4D-CTH + LSC groups were analyzed by HE staining at the end of the observation period. Notably, no significant difference was observed in histology between the two groups (Figure 7B). Therefore, we believed that the 4D-CTH scaffold exhibited no biological toxicity toward various organs and speculated that the 4D-CTH scaffold was a bioengineering scaffold without biological toxicity.

## 4 Discussion

Diabetic keratopathy is a common complication of diabetes. Owing to the accumulation of peripheral blood vessels and nerves because of local high sugar levels, diabetic wounds are difficult to heal, and such patients are prone to serious infections and may even face the risk of amputation, resulting in a huge physical and mental burden (Stuard et al., 2020). Furthermore, long-term hyperglycemia can lead to the accumulation of advanced glycosylation end products such as reactive oxygen species, causing vascular injury, impaired vascular endothelial integrity, and insufficient oxygen supply to these wounds. Additionally, high glucose levels can also lead to local bacterial reproduction, thus interfering with wound healing. Conversely, continuous oxidative stress causes the aging of fibroblasts, endothelial cells, keratinocytes, and MSCs, affecting the formation of tissues, blood vessels, and the epithelium. Simultaneously, the accumulation of glucose metabolites in these wounds can glycosylate some pro-inflammatory factors and chemokines, resulting in reduced cytokine activity and hindering wound healing (Ren et al., 2022; Ju et al., 2023).

Currently, the clinical treatment of diabetic keratopathy includes blood glucose control, surgical debridement, antibiotic therapy, and local wound dressing change. However, long-term, repeated surgical debridement can cause great pain and financial burden to patients. The emergence of resistant bacteria can affect the effectiveness of antibiotics (Priyadarsini et al., 2020). Furthermore, traditional dressings have poor skin adhesion and need to be replaced frequently. Studies have shown that stem cells play an important role in promoting wound healing (Ju et al., 2023); thus, strategies based on stem cells should be considered as alternatives. (Gardikiotis et al., 2022).

MSCs are a group of cells with the ability of self-renewal and multidirectional differentiation (Sun et al., 2022). MSCs play an important role in tissue repair and regenerative medicine (Weng et al., 2021). Furthermore, they can migrate to damaged tissue sites and differentiate into specific cells. Additionally, MSCs can also secrete various cytokines and growth factors to promote tissue regeneration (Wang et al., 2023a). Herein, the diabetic rabbits were considered as research objects. LSCs can be derived from diverse sources, extracted easily, and differentiated into corneal epithelial cells (Bonnet et al., 2021). A variety of cell types can be used to differentiate into LSCs, such as embryonic stem cells, induced pluripotent stem cells, adipose-derived mesenchymal stem cells, bone marrow mesenchymal stem cells, etc (Wang et al., 2023b). Currently, the clinical acquiescence of stem cell therapy mostly uses local direct injection. However, when the wound injury is large, direct injection will lead to a low local cell retention rate, reduce the biological activity of MSCs, and hinder the effect of stem cell therapy (Gill et al., 2018; Chen et al., 2023). Recent studies have shown that hydrogels have good biocompatibility and unique micropore structure and are good carriers for drugs and cells (Wang et al., 2020; Zhao et al., 2023). However, some traditional hydrogels usually have submicron or nano-pore size, and cells in the nano-pore hydrogels are difficult to seed and exert an effect on the cell morphology, which limits the growth and proliferation of cells to a certain extent (Ma et al., 2022). 4D bioprinting technology includes a time dimension on the basis of 3D bioprinting, i.e., over time, the carrier can be deformed, overcoming the problems that traditional 3D printing cannot fit the wound well and the traditional hydrogel aperture is uneven (Pradhan et al., 2020).

Herein, an LSC vector with a uniform aperture and adjustable shape was prepared using natural marine extract and 4D printing technology, and the performance indices of the vector were comprehensively evaluated by performing a series of experiments. The results showed that the performance indices of the vector were better than those of the traditional vector, and the cell transfer and local cell survival rates were significantly improved. During corneal wound healing, LSCs proliferated and differentiated into corneal epithelial cells and migrated to the center, which was a key process in wound healing. Animal experiments confirmed that LSCs transplanted with this carrier significantly promoted wound healing. HE and immunofluorescence staining showed that compared with the PBS group, the repair effect of LSCs was significantly improved, which might be related to the uniform pore size of 4D-CTH, the local retention rate of LSCs, and the moist and suitable microenvironment for the growth and proliferation of LSCs. Thus, in addition to repairing corneal epithelial injury, the 4D-CTH scaffold can also repair skin, diabetic foot, cervical erosion, and other tissue injuries, which has broad application prospects.

## Data Availability

The raw data supporting the conclusion of this article will be made available by the authors, without undue reservation.
