# Supplementary material for: Limbal stem cells carried by a four-dimensional -printed chitosan-based scaffold for corneal epithelium injury in diabetic rabbits
Source: Front Physiol. 2024 Jun 3;15:1285850. doi: 10.3389/fphys.2024.1285850 (PMC11180886; doi:10.3389/fphys.2024.1285850)
Supplement: Supplementary file 1 [file DataSheet1.docx]

Supplementary Material


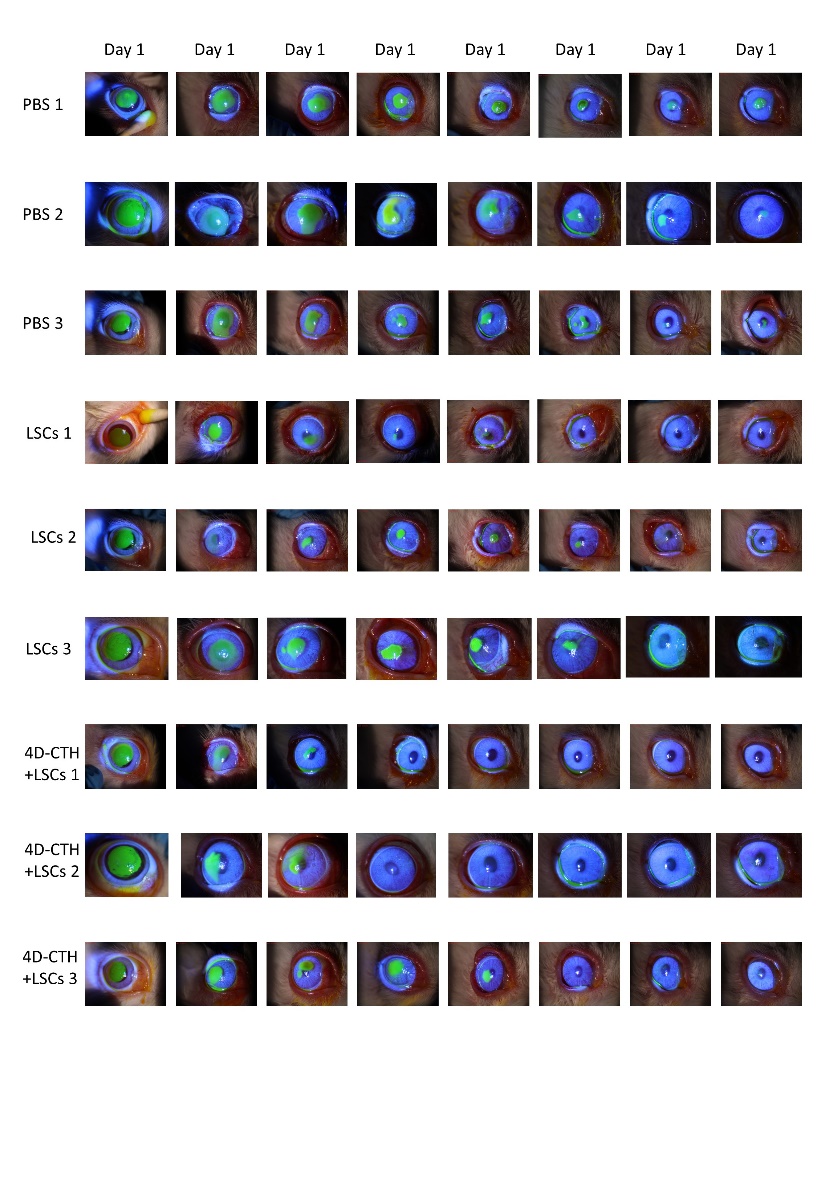


Supplementary Figure 1. Cobalt blue light images of wound healing under different treatment conditions at 1-7 days of wound healing.


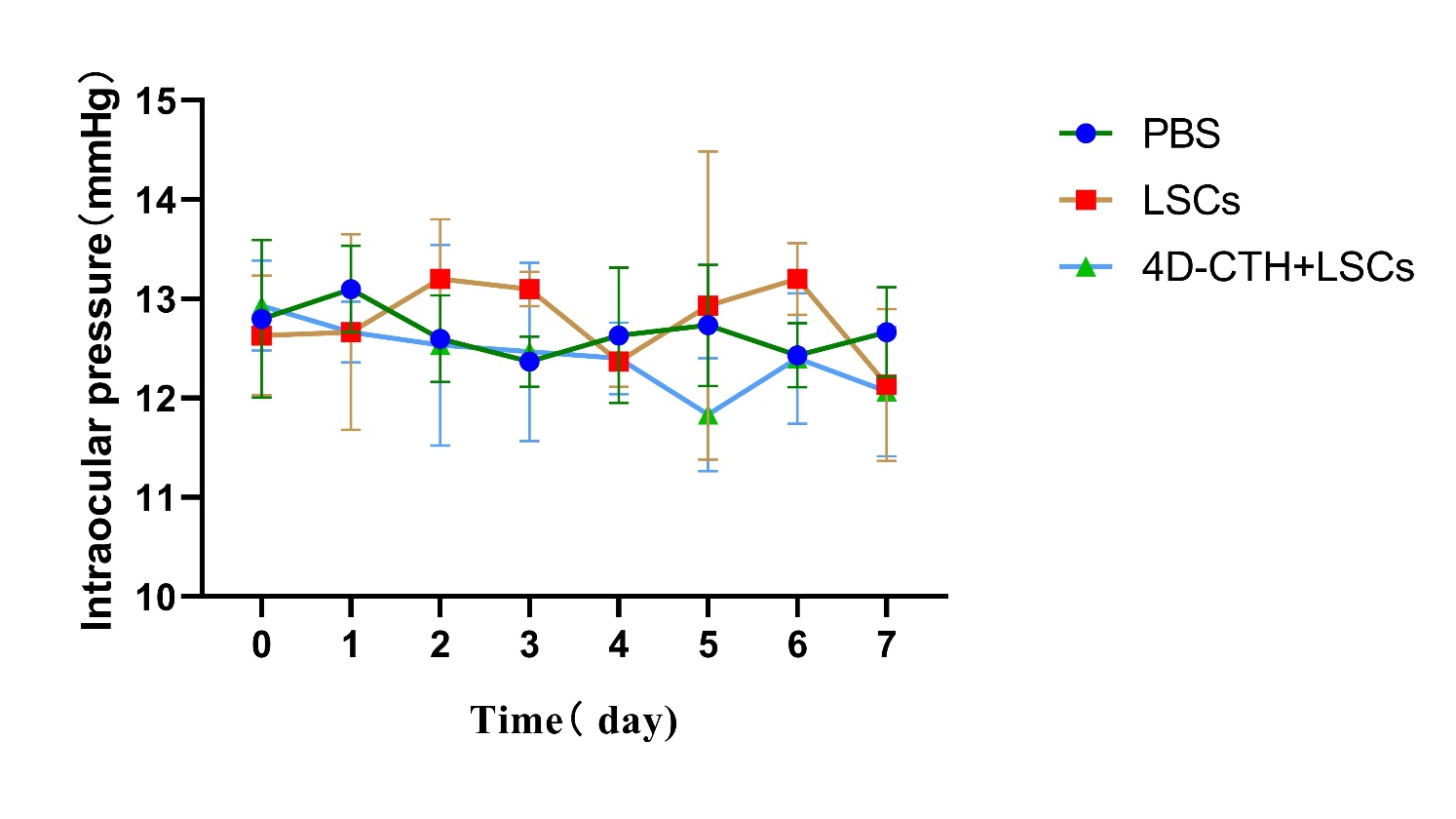


**Supplementary Figure 2.** Results of the statistical analysis of the IOP at 0-7 days of wound healing under different treatment conditions.
